# Incidence and spread pattern of lymph node metastasis from submandibular gland cancer

**DOI:** 10.1007/s00405-023-08020-x

**Published:** 2023-05-22

**Authors:** Kotaro Tamagawa, Naoki Otsuki, Hikari Shimoda, Naruhiko Morita, Tatsuya Furukawa, Masanori Teshima, Hirotaka Shinomiya, Ken-ichi Nibu

**Affiliations:** 1 Department of Otolaryngology-Head and Neck Surgery, Kobe University Graduate School of Medicine, 7-5-1, Kusunoki-Cho, Chuo-Ku, Kobe, Hyogo 650-0017 Japan; 2 Present Address: Department of Otolaryngology-Head and Neck Surgery, Hyogo Prefectural Harima-Himeji General Medical Center, 3-264, Kamiya-Cho, Himeji, Hyogo 670-8560 Japan

**Keywords:** Submandibular gland cancer, Cervical lymph node, Neck dissection, Occults metastasis

## Abstract

**Objectives:**

To clarify the indication of neck dissection (ND) for patient with submandibular gland (SMG) cancer.

**Methods:**

A total of 43 patients with SMG cancer were retrospectively analyzed. Forty-one patients underwent ND: Levels I–V in 19 patients, Levels I–III in 18 patients, and Level Ib in 4 patients. The other two patients did not undergo ND, since preoperative diagnoses were benign. Postoperative radiotherapy was performed in 19 patients with positive surgical margin, high grade cancer or stage IV disease.

**Results:**

LN metastases were pathologically confirmed in all patients with cN + and 6 out of 31 patients with cN–. No patients developed regional recurrence during follow-up periods. Ultimately, LN metastases were pathologically confirmed in 17 of 27 high grade, one out of 9 intermediate grade, but not in 7 low grade.

**Conclusions:**

Prophylactic neck dissection should be considered in T3/4 and high grade SMG cancers.

## Introduction

Salivary gland cancers represent about 6% of all head and neck malignant neoplasms, comprising various pathological types with differing biological behavior [1]. About 10% of all salivary gland neoplasms arise in SMG and 30–50% of them were malignant [2, 3]. Radical resection followed by postoperative radiotherapy is a mainstay treatment for patients with high-risk features (advanced clinical T stage, pathological high grade, or a positive or close surgical margin) to improve loco-regional control [2, 3]. Metastasis to cervical lymph nodes (LN) has been reported as a major prognostic factor for patients with SMG cancer [4]. Reported rate of occult LN metastasis in submandibular cancers ranged from 15 to 48% and high-grade histological type and advanced clinical stage have been reported to be associated with nodal disease [5, 6]. However, indications and extents of neck dissection (ND) for SMG cancer have not been well-defined due to its low incidence. The aim of this study was to clarify the risk factors of LN metastasis and identify appropriate indication and extent of neck dissection in individual patients with SMG cancers.

## Materials and methods

### Patients

Medical records of 43 patients who had radical resection of histologically confirmed SMG cancer between January 1994 and December 2018 were retrospectively reviewed. Two patients with lung metastasis at the time of initial diagnosis were included in this analysis, since lung metastases were small and we considered that loco-regional control by surgical resection would improve the quality of life of these patients. The clinical characteristics of 43 patients are outlined in Table 1. The median age was 68 years ranging from 18 to 93 years. The pathological grade was classified as high grade in 27 patients, intermediate grade in 9 patients, and low grade in 7 patients according to WHO classification [1]. The most common pathological type was adenoid cystic carcinoma (AdCC) (12), followed by mucoepidermoid carcinoma (MEC) (9), carcinoma ex pleomorphic adenoma (CxPA) (8), salivary duct carcinoma (SDC) (5), squamous cell carcinoma (SCC) (3), adenocarcinoma NOS (3), myoepithelial carcinoma (1), basal cell adenocarcinoma (1) and undifferentiated carcinoma (1).

**Table 1:** Clinical features of the 43 patients

Clinical features	*n*	(%)
Gender		
Male	25	(58)
Female	18	(42)
Age (years)		
< 65	16	(37)
≧65	27	(63)
Clinical T		
T1	4	(9)
T2	12	(28)
T3	19	(44)
T4	8	(19)
Clinical N		
N0	31	(72)
N1	1	(2)
N2a	0	(0)
N2b	11	(26)
Clinical M		
M0	41	(95)
M1	2	(5)
Clinical Stage		
I	4	(9)
II	11	(26)
III	11	(26)
IV A	15	(35)
IV B	0	(0)
IV C	2	(5)
Histology		
Adenoid cystic carcinoma (AdCC)	12	(28)
Mucoepidermoid carcinoma (MEC)	9	(21)
Carcinoma ex pleomorohic adenoma (CXPA)	8	(19)
Salivary duct carcinoma (SDC)	5	(12)
Squamous cell carcinoma (SqCC)	3	(7)
Adenocarcinoma, NOS (NOS)	3	(7)
Myoepithelial carcinoma	1	(2)
Basal cell adenocarcinoma	1	(2)
Undifferentiated carcinoma	1	(2)
Total	43	

SMG tumors were assessed by fine-needle aspiration cytology (FNAC) under ultrasonography (US) in addition to diagnostic imaging, such as US, computed tomography (CT), and magnetic resonance imaging (MRI). The FNAC results were classified into the following five diagnostic categories: malignancy, suspected malignancy, undetermined, benign lesion, and insufficient material for analysis. Of the 43 patients, two were diagnosed with benign lesions preoperatively and SMG cancer by histopathological examination of resected permanent specimens.

The extent of diseases was determined by physical and endoscopic examinations, MRI, CT, fluorodeoxyglucose–positron emission tomography (PET) and/or gallium scintigraphy. In general, preoperative evaluation of clinical lymph node metastasis was performed by contrast-enhanced CT. A positive lymph node was defined as an enhanced node measuring 10 mm or more in minimum diameter. The extent of resection was determined by intraoperative rapid diagnosis.

All cancers were retrospectively restaged according to 8th UICC TNM staging system [7]. T stage was determined as T1 in 4 patients, T2 in 12 patients, T3 in 19 patients, and T4 in 8 patients. N stage was determined as N0 in 31 patients, N1 in one patient and N2b in 11 patients. Patients were followed up at least 3 years or until they died. The median follow-up period was 57 months ranging from 5 to 194 months.

This study was approved by the Institutional Review Board of Kobe University Graduate School of Medicine before the collection of patient information (No. 2910). Risk factors for pathological metastasis were determined using the chi-square test. EZR version 4.2.1 was used for the statistical analyses [8]. Overall survival rates were evaluated using the Kaplan–Meier method. A *p* value of < 0.05 indicated statistical significance.

### Treatment of primary site and neck

**Fig. 1:**
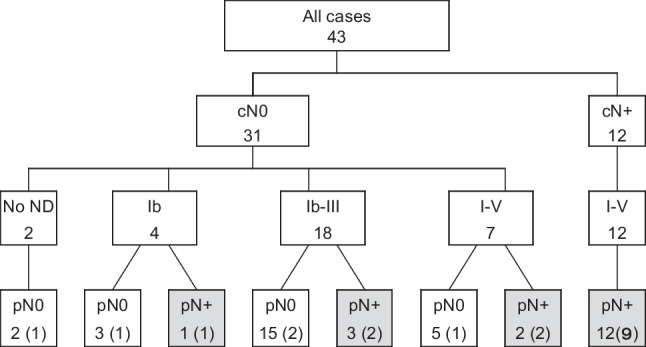
Therapeutic patterns and pathological neck metastasis. (Numbers in parentheses indicate patients who received postoperative radiotherapy)

The extent of resection was dependent on the location and extent of the tumor, and the involvement of the lingual nerves and hypoglossal nerves. In principle, we performed modified radical neck dissection (MRND) in 12 patients with clinically positive neck (cN+) and prophylactically in 6 patients with cT3/T4 disease and 1 patient with T1/2 high grade disease (Fig. 1). Supraomohyoid neck dissection (SOND) was performed in 18 patients with cT3T4/cN–. Selective dissection of level Ib was performed in 4 patients with cT2T3/cN– or low-grade disease. Two patients did not undergo neck dissection, since preoperative diagnoses were benign. In 19 patients with positive surgical margin, stage IV or high-grade disease, or multiple LN metastases, postoperative radiotherapy (PORT) was administered with a daily dose of 2.0 Gy × 5 days per week for a total of 50–70 Gy.

## Results

All 12 patients with clinical positive LN metastasis (cN+) had pathologically positive LN metastasis (pN+). In patients with cN+/pN+, LN metastasis were distributed in submandibular area (level Ib) in 9 patients (75%), level Ia in one patient (8%), level IIa in 10 patients (83%), level IIb in 2 patients (17%), level III in 10 patients (83%), level IV in 4 patients (33%), and level V in 5 patients (42%) (Table 2). Six out of 31 patients with clinical negative LN metastasis (cN–) had pathologically positive LN metastasis. All LN metastases were limited in levels I and II (Table 2). Among the 43 patients, local recurrence occurred in 6 patients, but no patients developed regional recurrence during follow-up periods. Thus, ultimately, 18 out of 43 patients with SMG cancer had pathological LN metastasis.

**Table 2:** Distribution of neck lymph node metastasis

	Ia (%)	Ib (%)	IIa (%)	IIb (%)	III (%)	IV (%)	V (%)
cN + /pN + *n* = 12	1 (8)	9 (75)	10 (83)	2 (17)	10 (83)	4 (33)	5 (42)
cN - /pN + *n* = 6	1 (17)	3 (50)	3 (50)	1 (17)	0	0	0

Incidences of clinical and pathological LN metastases according to T stage are summarized in Table 3. Among the 31 patients with cN–, pathologically positive LN metastasis were observed in 5 out of 6 patients with cT3/4 disease and in one patient with T2 SDC. Five out of 6 patients with cN-/pN+ were diagnosed as having high-grade cancer and intermediate grade cancer as described below and eventually died of disease.

**Table 3:** T stage and neck lymph node metastasis

cT	*n*	pN +	cN –/pN + (%)	cN + /pN + (%)
1	4	0 (0)	0 (0)	0 (0)
2	12	2 (17)	1 (8)	1 (8)
3	19	12 (63)	3 (16)	9 (47)
4	8	4 (50)	2 (25)	2 (25)
Total	43	18	6	12

Incidences of pathologically positive LN metastasis according to the histological types are summarized in Table 4. According to the histological grades, pathological LN metastases were observed in 63% (17/27) in high grade, 11% (1/9) in intermediate grade, and 0% (0/7) in low grade. Pathological types of the 6 patients with cN–/pN + were high-grade mucoepidermoid carcinoma (1), squamous cell carcinomas (1), high-grade adenoid cystic carcinomas (2), and myoepithelial carcinoma (1).

**Table 4:** Pathological types and neck lymph node metastasis

Pathological types	*n*	pN + (%)	Occult metastasis (%)
Low grade	7	0 (0)	0
MEC—low	4	0	0
CXPA- low	2	0	0
Basal cell adenocarcinoma	1	0	0
Intermediate grade	9	1 (11)	1
AdCC—intermediate	7	0	0
MEC- intermediate	1	0	0
Myoepithelial carcinoma	1	1 (100)	1 (100)
High grade	27	17(63)	5 (19)
CXPA- high	6	2 (33)	0
AdCC—high	5	4 (80)	2 (40)
SDC	5	4 (80)	1 (20)
MEC- high	4	3 (75)	1 (25)
SqCC	3	1 (33)	1 (33)
Adenocarcinoma NOS	3	2 (67)	0
Undifferentiated carcinoma	1	1 (100)	0
Total	43	18 (40)	6 (14)

Frozen sections of level II lymph node were performed on 14 patients at the time of surgery. The intraoperative diagnosis was positive in one patient with cN+ adenoid cystic carcinoma but negative in the other 13 patients. However, metastasis to other level II lymph nodes was pathologically confirmed in 3 of the 12 patients at the final diagnosis.

Indications of PORT in 19 patients are shown in Table 5. One patient who had stage IV high-grade cancer did not receive PORT due to previous history of radiotherapy to the neck. In addition, four patients with low-grade mucoepidermoid carcinoma or low-grade adenoid cystic carcinoma did not receive PORT in spite of close surgical margin. Among them, one patient had local recurrence, which was successfully salvaged by surgery and PORT. Other 12 patients with Stage IV and/or high grade refused PORT mostly due to advanced age. Among them, 3 patients developed local relapse and died of disease.

**Table 5:** Indication criteria of postoperative radiotherapy

Criteria	PORT ( +)	PORT (–)
Stage IV, positive surgical margin, high grade	9	0
Stage IV, high grade	3	4 (1)
Positive surgical margin	3	4
Positive surgical margin, high grade	2	0
High grade	2	6
Stage IV	0	2
None	0	8
Total	19	24

*PORT* Postoperative radiotherapy, () Previous RT for other disease

**Fig. 2:**
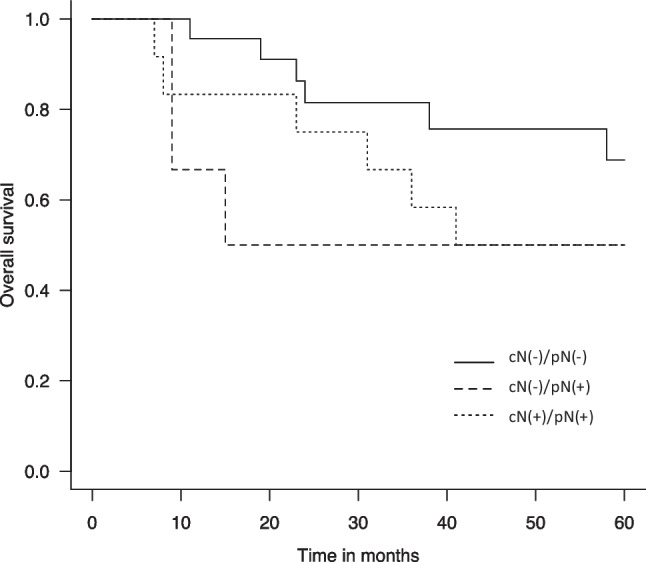
Kaplan–Meier analysis of overall survival according to clinical (cN) and pathological (pN) neck metastasis status

All 43 patients had a 5-year overall survival (OS) rate of 64%. According to lymph node metastasis status, those with cN–/pN–, cN–/pN+, and cN+/pN+ had 5-year OS rates of 68.8%, 50.0%, and 50.0%, respectively (Fig. 2). There is statistically significant between cN–/pN– and cN–/pN+ (*p* = 0.019). Pathological lymph node metastasis in the patients with clinically negative node was identified as a significant prognostic factor for SMG cancer.

## Discussion

Although tumors of SMG are uncommon, up to half of them are malignant [3]. According to Reports of Head and Neck Cancer Registry of Japan (HNCR) conducted by Japan Society for Head and Neck Cancer, 24% (714/2928) of major salivary gland cancers originated in SMG between 2013 and 2017[9]. The most common pathological type was AdCC (28%), followed by SDC (19%), CxPA (10%), Adenocarcinoma NOS (9%), SCC (8%), and MEC (7%). In consistent with the present study, more than half were assumed as high grade. Considering the high probability of high-grade cancers and difficulty in making accurate preoperative diagnoses with fine needle aspiration cytology due to the variety of histological types of salivary gland cancers, surgical procedures should be planned in case of high-grade cancers. As for cervical lymph node, 37% (265 patients) presented with clinical LN metastases, 65% (463 patients) underwent some type of ND (Total ND: 141, Selective ND: 307) and 42% (297patients) were treated with radiotherapy in the same report [9]. While minute data are not available due to the nature of the registry, these data suggest that 37% of the patients underwent therapeutic ND and about a quarter of patients underwent prophylactic ND.

A recent study reported that pN+ was confirmed in all neck levels (Level I to V) of the patients who presented with cN+ [10]. Accordingly, all patients with cN+ had pN+ in the present series. LN of levels Ia, Ib, IIa, IIb, III, IV and V were pathologically positive in 8%, 75%, 83%, 17%, 83%, 33% and in 42%, respectively. Thus, the present results support our policy that levels I to V should be dissected in therapeutic ND for the clinically positive neck.

The incidence of pathologically positive LN metastasis in the patients with cN– in our series was as low as 19% in accordance with the previous reports. To date, there is no consensus regarding indications for prophylactic ND and the extent of patients with SMG cancer. High-grade pathological types and tumor size more than 4 cm have been reported as predictive factors for occult LN metastasis [11]. In our present study, cT3/T4 and pathological high grade were significant risk factors for pathological lymph node metastasises in the neck (Table 6). In addition, the distribution of occult lymph node metastasis was limited to level I/II in accordance with the previous report, supporting the prophylactic neck dissection including levels I/II for the patients with cT3/T4 and/or high-grade pathological type [12].

**Table 6:** Risk factors of pathological lymph node metastasis

Risk factors	pN –	pN +	*p*
Age			
< 65	9	7	0.84
≧65	16	11	
T classification			
T1, T2	14	2	< 0.01
T3, T4	11	16	
Pathological grade			
Low, int	15	1	< 0.01
High	10	17	

Pathologically high-grade cancers including high-grade mucoepidermoid carcinoma, salivary duct carcinoma, squamous cell carcinoma, adenocarcinoma and undifferentiated carcinoma have been reported to be at more than 50% risk of neck metastasis [13]. Thus, the risk of occult nodal disease is high enough to warrant END. However, it is hard to predict the accurate histological type and grade despite of the development of FNAB and imaging [14]. Therefore, it is not practical to determine the indication of prophylactic ND on the basis of histological grade at present. In our series, 4 patients underwent open incisional biopsy to assess pathological diagnosis. All four patients were diagnosed as malignant tumors, but an accurate histological type was obtained in only one patient with adenoid cystic carcinoma. Recently, it was reported by a meta-analysis that ultrasound-guided core needle biopsy of salivary glands was an excellent diagnostic tool in terms of accuracy and safety profile [15, 16]. In the future, this diagnostic tool should be considered in selected cases.

The drainage route of lymph from the SMG is carried to the adjacent submandibular nodes located on the bony, deep, and cutaneous surfaces of the gland. The efferent lymph flow from the submandibular lymph nodes usually follows the main (internal jugular lymph nodes) and anterior accessory pathway [17]. Han et al. reported that 85% of pN+ submandibular cancer cases were found with Level IIa metastasis, as shown in our series (83%) [13]. Taken together, the presence of positive Level IIa lymph node metastasis may necessarily indicate a risk of lateral neck lymph node metastasis. Although some studies support an intraoperative rapid diagnosis of Level II lymph node by frozen section, our results showed low sensitivity (50%) and high frequency of false negative (50%) [18]. Therefore, decision making of therapeutic neck dissection by the intraoperative rapid diagnosis of Level II lymph node is not reliable so far.

Postoperative radiotherapy (PORT) prevents nodal relapses for selected patients at high risk for regional failure. In this study, we selected PORT for the patients with high risk features, such as Stage lV, pathologically positive surgical margin and high pathological grade. PORT may improve neck control [19, 20].

This study has several limitations. First, we included a relatively small number of patients with submandibular gland cancers who were treated with surgical resection in one institution. We believe that a future study should include a larger patient population by multicenter joint research due to relatively rare diseases. Second, the retrospective nature of this analysis introduces some selection biases. The difficulty of preoperative diagnosis leads to incomplete resection of primary tumor followed by adjuvant radiotherapy. We should have greater uniformity of the treatment approach, which will lead to more robust observations. Finally, the mean overall follow-up of approximately 5 years might be considered relatively short in the setting of low grade or intermediate malignancy (mucoepidermoid carcinoma and ACC), and thus we believe that long-term observation is necessary.

## Conclusions

In the treatment of submandibular cancer, MRND (Level I to V) is recommended for clinically positive neck. Prophylactic ND is strongly recommended for patients with cT3/T4. Prophylactic ND should include at least levels I and II.

## Data Availability

The data that support the findings of this study are not openly available due to reasons of sensitivity and are available from the corresponding author, [N,O], upon reasonable request.
